# Testing the Utility of Polygenic Risk Scores for Type 2 Diabetes and Obesity in Predicting Metabolic Changes in a Prediabetic Population: An Observational Study

**DOI:** 10.3390/ijms232416081

**Published:** 2022-12-16

**Authors:** Felipe Padilla-Martinez, Łukasz Szczerbiński, Anna Citko, Marcin Czajkowski, Paulina Konopka, Adam Paszko, Natalia Wawrusiewicz-Kurylonek, Maria Górska, Adam Kretowski

**Affiliations:** 1 Clinical Research Centre, Medical University of Bialystok, 15-276 Bialystok, Poland; 2 Department of Endocrinology, Diabetology and Internal Medicine, Medical University of Bialystok, 15-276 Bialystok, Poland; 3 Faculty of Computer Science, Bialystok University of Technology, Wiejska 45a, 15-351 Bialystok, Poland; 4 Department of Clinical Genetics, Medical University of Bialystok, 15-276 Bialystok, Poland

**Keywords:** polygenic risk scores, type 2 diabetes, prediabetes, obesity, prediction, genomics

## Abstract

Prediabetes is an intermediate state of hyperglycemia during which glycemic parameters are above normal levels but below the T2D threshold. T2D and its precursor prediabetes affect 6.28% and 7.3% of the world’s population, respectively. The main objective of this paper was to create and compare two polygenic risk scores (PRSs) versus changes over time (Δ) in metabolic parameters related to prediabetes and metabolic complications. The genetics of 446 prediabetic patients from the Polish Registry of Diabetes cohort were investigated. Seventeen metabolic parameters were measured and compared at baseline and after five years using statistical analysis. Subsequently, genetic polymorphisms present in patients were determined to build a T2D PRS (68 SNPs) and an obesity PRS (21 SNPs). Finally, the association among the two PRSs and the Δ of the metabolic traits was assessed. After a multiple linear regression with adjustment for age, sex, and BMI at a nominal significance of (*p* < 0.05) and adjustment for multiple testing, the T2D PRS was found to be positively associated with Δ fat mass (FM) (*p* = 0.025). The obesity PRS was positively associated with Δ FM (*p* = 0.023) and Δ 2 h glucose (*p* = 0.034). The comparison of genotype frequencies showed that AA genotype carriers of *rs10838738* were significantly higher in Δ 2 h glucose and in Δ 2 h insulin. Our findings suggest that prediabetic individuals with a higher risk of developing T2D experience increased Δ FM, and those with a higher risk of obesity experience increased Δ FM and Δ two-hour postprandial glucose. The associations found in this research could be a powerful tool for identifying prediabetic individuals with an increased risk of developing T2D and obesity.

## 1. Introduction

Prediabetes is an intermediate state of hyperglycemia with glycemic parameters above normal but below the diabetes threshold [1]. Clear links between cardiovascular disease, metabolic syndrome, and prediabetes have emerged in recent years. Nevertheless, the pathophysiological defects seen in prediabetes can be managed by lifestyle modifications [2]. Type 2 diabetes (T2D) remains a significant clinical burden worldwide and is costly for individuals, healthcare systems, and economies [3]. T2D and its precursor, prediabetes, affect 6.28% and 7.3% of the world’s population, respectively [4]. The pathophysiological defects of T2D have been recognized in prediabetic cohorts, some of which are alpha and beta cell dysfunction, insulin resistance, increased lipolysis, and inflammation [5,6]. Furthermore, recent studies have given insight into long-term complications of diabetes and cardiovascular diseases and how they could manifest in some people with prediabetes [7,8]. According to the Polish Diabetes Association and American Diabetes Association, prediabetes is defined by the presence of impaired fasting glucose (IFG) and/or impaired glucose tolerance (IGT) and/or HbA1C (5.7–6.4%). IFG is defined as fasting plasma glucose levels from 100 to 125 mg/dL (from 5.6 to 6.9 mmol/L) and IGT as 2 h plasma glucose levels during 75-g OGTT from 140 to 199 mg/dL.

Obesity is a global pandemic; over one billion people will be obese worldwide by 2030 [9]. Research shows that obesity is a common risk factor for the development of prediabetes and T2D [10]. Moreover, in the last years, the highly potential causal effect of obesity on prediabetes and the key role of adipose tissue in insulin resistance has been discovered [11]; therefore, finding associations between them and a possible genetic link will be of great interest.

The advent of genotyping and sequencing technologies has contributed to the discovery of many genetic variants contributing to T2D pathogenetic complexity. Likewise, the generation of genome-wide variation data has become common for predicting metabolic diseases [12]. T2D has well-established risk loci and likely contains many genetic determinants with effects too small to be detected at genome-wide levels of statistical significance [13]. This demonstrates that all common variants across the genome explain a much higher proportion of heritability in many complex traits than can be seen using only a small subset of significant single nucleotide polymorphisms (SNPs) [14]. An approach to converting genetic data to a predictive measure of disease susceptibility is to add the risk effects of loci into a single genetic risk score called the polygenic risk score (PRS) [15]. The number of studies combining phenotypic and genetic variables to predict diabetes risk has increased recently, and they show generally promising results. In the last decade, studies developing PRSs for diabetes have been increasing, as stated in a 2020 systematic review; fourteen studies were analyzed and their areas under the curve compared [16,17], showing that their accuracy was high enough to discriminate patients and controls [18,19], thus demonstrating their risk prediction power and their clinical relevance. PRSs provide support for diagnosis decisions and can reliably discriminate among diabetes subtypes [20,21]. The use of T2D PRSs for predicting the risk of other diabetic pathologies, such as cardiovascular [8], renal complications [22], and transplant setting [23], has been studied and discussed in recent years.

A proof-of-concept study identified six distinct clusters of sub-phenotypes for prediabetes. Of the identified groups, two have shown imminent diabetes risks [24]. This demonstrates that pathophysiological heterogeneity exists before the diagnosis of T2D. In addition, patients with prediabetes can suffer from coronary artery disease, obesity, and diastolic heart failure even before progressing to overt diabetes. However, prediabetes can be controlled by a change of habits. With this knowledge, it is essential to identify prediabetic patients with a higher risk of developing T2D and obesity in order to take appropriate measures to optimize glycemic control. To address this issue, the current paper proposes an approach for associating genetic information and metabolic traits related to T2D and metabolic complications in a prediabetic population. A tool that can identify individuals with increased risk could prevent T2D development and other complications, thus improving precision medicine in diabetes.

## 2. Results

### 2.1. Subjects’ Characteristics and Metabolic Parameters at Baseline and Follow-Up

The baseline characteristics of the 446 participants are summarized in Table 1. Approximately half of the participants were female, and the median age was 42.54 years. The median BMI was 26.87 kg/m^2^, the median fasting glucose (IFG) was 101 mg/dL, and the median HbA1C was 5.76%, which aligns with the diagnostic criteria of prediabetes required for recruitment. The description and comparison of the metabolic parameters at baseline and follow-up of the prediabetic group are presented in Table 1. After the adjustment of the *p*-value, the variables in the population that did not present statistically significant differences are Chol, Tg, Hgl, and Ldl. The variables that have statistically significant differences (in order from highest to lowest) are fasting glucose, SF, Age, FM, IPAQ, FFM, HbA1c, VF, 2 h glucose, VAT/SAT ratio, BMI, fasting insulin, MM, and 2 h insulin.

### 2.2. Construction of Polygenic Scores for T2D

**Figure 1:**
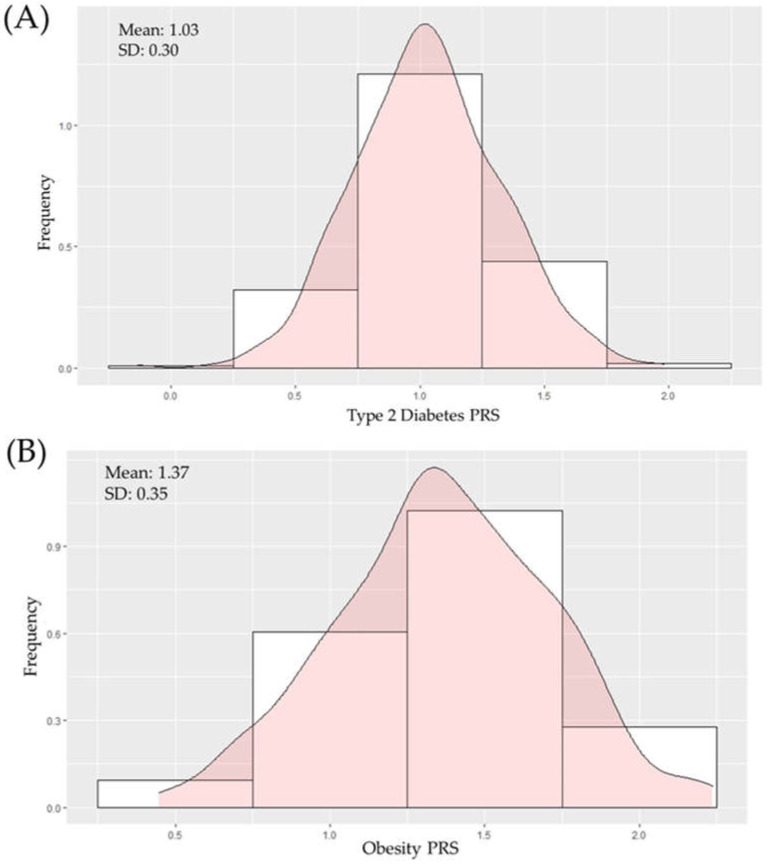
Distribution of polygenic risk scores (PRSs) for T2D (**A**) and obesity (**B**) across 446 prediabetic individuals in PolRed.

The mean for the T2D PRS in the prediabetic cohort was 1.03 (range: 0.23–1.64), with an SD of 0.30 (Figure 1A). The mean obesity PRS in the prediabetic cohort was 1.37 (range: 0.45–2.24), with an SD of 0.35 (Figure 1B).

### 2.3. Association between T2D PRS and Metabolic Parameters

The T2D PRS was associated with baseline values of BMI, FFM, FM, fasting glucose, and fasting insulin at a nominal significance of *p* < 0.05 after adjustment for multiple testing (Supplementary Table S3). Table 2 shows the associations among the T2D PRS and the changes in metabolic parameters after follow-up. The T2D PRS was associated with Δ FM at a nominal significance of *p* < 0.05 after adjustment for multiple testing. As the beta coefficient is positive, for every SD increasing in the PRS, Δ FM will increase 0.0049 kg, with an adjusted r-squared value of 0.10369. After the testing, a significant association was not found between T2D PRS and Δ in FFM, MM, VF, SF, VAT/SAT ratio, IPAQ, fasting glucose, 2 h glucose, HbA1c, fasting insulin, 2 h insulin, Chol, Tg, Hdl, or Ldl. The T2D PRS was associated with Δ FM for the female and the male groups in the gender stratification analysis, at a nominal significance of *p* < 0.05 after adjustment for multiple testing (Supplementary Table S5).

### 2.4. Association between Obesity PRS and Metabolic Parameters

The Obesity PRS was only associated with the baseline value of BMI at a nominal significance of (*p* < 0.05) after adjustment for multiple testing (Supplementary Table S4). Table 3 summarizes the associations between the obesity PRS and changes in metabolic parameters after the follow-up. A high obesity PRS was associated with Δ FM and Δ 2 h glucose at a nominal significance of *p* < 0.05 after adjustment for multiple testing. As their beta coefficients are positive, for every increase in SD in the PRS, Δ FM increased by 0.0056 kg and Δ glucose after 120 min increased by 0.0013 mg/dl, with an adjusted r-squared value of 0.21025. After testing, obesity PRS was not significantly associated with Δ FFM, MM, VF, SF, VAT/SAT ratio, IPAQ, fasting glucose, HbA1c, fasting insulin, 2 h insulin, Chol, Tg, Hdl, or Ldl. The Obesity PRS was associated with Δ FM and Δ 2 h glucose for the female and the male groups on the gender stratification analysis, at a nominal significance of *p* < 0.05 after adjustment for multiple testing (Supplementary Table S6).

### 2.5. Association of Genotypes’ Frequencies with Changes in Metabolic Parameters

Tests were performed to check whether the genotypes’ frequencies significantly affected all 17 metabolic parameters. All the SNPs included in the T2D PRSs and obesity PRSs (69 SNPs in total) were analyzed, and the metabolic parameters were stratified by investigated genotypes with a significant association or a tendency (Table 4). No significant deviation from the Hardy–Weinberg equilibrium was reported for any of the investigated SNPs.

It was observed that AA genotype carriers of rs10838738 were significantly higher in Δ 2 h glucose and in Δ 2 h insulin (Table 4). No other significant differences were observed among the other genotypes; however, a tendency toward a lower Δ FM and Δ VF was noticed in GG genotype carriers of rs2260000. Among carriers of investigated genetic variants in rs7647305, a tendency in Δ 2 h glucose and Δ IPAQ was noticed. Another tendency toward a lower Δ 2 h glucose in GG genotype carriers of rs29941 was noticed (Table 4).

**Figure 2:**
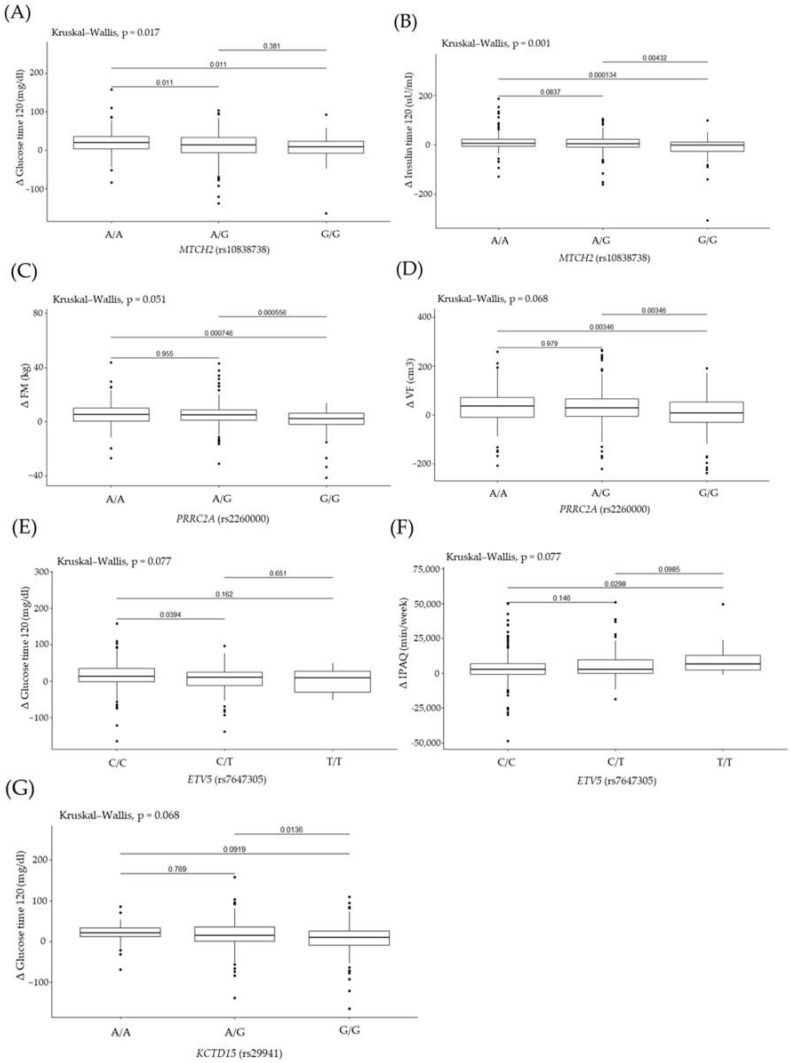
Association of genotype rs10838738 with (**A**) Δ 2 h glucose (mg/dL) and (**B**) Δ 2 h insulin (uU/mL). Genotype rs2260000 with (**C**) Δ FM (kg) and (**D**) Δ VF (cm3). Genotype rs7647305 with (**E**) Δ 2 h glucose (mg/dL) and (**F**) Δ IPAQ (min/week). Genotype rs29941 with (**G**) Δ 2 h glucose (mg/dL).

The AA genotype carriers of rs10838738 presented significantly greater Δ 2 h glucose (Figure 2A) than the AG and GG genotypes. The Δ in 2 h insulin (Figure 2B) had a significantly smaller difference when comparing the GG genotype and the AG and AA genotypes. By analyzing the differences among the rs2260000 genotypes, we observed that the GG genotype carriers presented a significantly lower Δ FM (Figure 2C) and Δ VF (Figure 2D) compared with the AA and AG genotype carriers. The CC genotype carriers of rs7647305 presented significantly greater Δ in 2 h glucose (Figure 2E) than CT genotypes. The Δ IPAQ (Figure 2F) had a significantly lower difference when comparing the CC genotype with the TT genotype. According to our analysis of the differences between rs29941 genotypes, AG genotype carriers presented significantly greater Δ in 2 h glucose (Figure 2G) than GG genotype carriers did.

## 3. Materials and Methods

### 3.1. Study Design and Participants

The study design of PolReD, with data from the 1000PLUS project merged into it, has been previously described [25,26]. Briefly, Caucasian participants were enrolled at the Department of Endocrinology, Diabetology and Internal Medicine, Medical University of Bialystok, Poland, between 2009 and 2012. Ethical approval for the study was originally obtained from the local ethics committee at the Medical University of Bialystok, Poland (R-I-002/436/2019). Before the study, all participants read and signed forms of informed consent. For this work, 446 prediabetic individuals were selected from the study population who, at baseline, had not been clinically diagnosed with T2D. Five years after the first examination, the same 446 subjects underwent a follow-up. To be included, patients had to be at high risk of developing diabetes, defined by the presence of impaired fasting glucose (IFG) and/or HbA1C. Patients with any infection, cardiovascular disease, recent surgery, or serious somatic disease were excluded. Patients with type 1 diabetes, type 2 diabetes, or latent autoimmune diabetes of adults were also excluded.

### 3.2. Sample Collection and Measurement

The patients fasted overnight and were asked to avoid intensive physical activity the day before the tests. Blood samples were collected from the whole blood of all participants during two visits: at baseline (Visit 1) and a follow-up after 5 years (Visit 2). Anthropometric measurements, including weight and BMI, were measured by standardized procedures. Biochemical measurements, including plasma glucose, serum triglycerides (Tg), total cholesterol (Chol), high-density lipoprotein (Hdl) cholesterol, and low-density lipoprotein (Ldl) cholesterol concentrations, were performed by the colorimetric method with Cobas c111 (Roche Diagnostics, Basel, Switzerland). Insulin concentration was measured in the serum using an immunoradiometric assay kit (DIAsource ImmunoAssays SA, Belgium). Glycated hemoglobin (HbA1C) was measured by the high-performance liquid chromatography method. Fasting glucose concentration and glucose concentration at two hours were measured in the plasma using the colorimetric method. The fat-free mass (FFM), fat mass (FM), muscle mass (MM), visceral fat (VF), subcutaneous fat (SF), and the ratio of visceral adipose tissue to subcutaneous adipose tissue (VAT/SAT) were measured by the multi-frequency bioimpedance method (MaltronBioScan 920-2, Maltron International Ltd., Rayleigh, UK). Physical activity was measured using the International Physical Activity Questionnaire (IPAQ). All the measurements were conducted using the same methodology for Visits 1 and 2.

### 3.3. Genotyping

DNA was extracted from the peripheral blood leukocytes using a classical salting out method. The SNPs were genotyped with TaqMan SNP technology from a ready-to-use human assay library (Applied Biosystems, MA, USA) using a high-throughput genotyping system, OpenArray (Life Technologies, CA, USA). A sample without a template was used as a negative control. The negative control was applicable in measuring any false positive signals caused by contamination.

### 3.4. Risk Score Analysis

PRSs were constructed for T2D, a common metabolic disorder characterized by chronic hyperglycaemia [27], and obesity, excessive, or abnormal accumulation of fat or adipose tissue in the body that may impair health [28]. Due to the limited availability of SNPs on our genotyping platform, we could include only a subset of the known genome-wide significant loci for T2D and obesity, resulting in a T2D PRS of 68 SNPs and an obesity score of 21 SNPs (Supplementary Tables S1 and S2). The software R was used to build the scores. Each PRS was calculated by summing the number of risk alleles carried (K = allele dosage for the SNP; 0, 1, or 2) by each individual, weighted by the effect size (β = per-allele log odds ratio for the minor alleles) estimates from well-established genome-wide associations (1) [29,30] selected from the Type 2 Diabetes Knowledge Portal [31].

(1) $$PRS=\sum_{i=0}^{n}\;\;\beta_{i}\;K_{i}$$

### 3.5. Statistical Analysis

The mean ± standard deviation (SD) or median (interquartile range) are reported for continuous normally or nonnormally distributed traits, respectively. Normality was assessed using the Shapiro–Wilk test. This analysis revealed that the studied parameters did not follow a normal distribution. Consequently, nonparametric tests were used for the statistical analysis between groups. The Wilcoxon signed-rank test was used to compare variables at baseline and follow-up. The change (Δ) in time (T2 minus T1) of each of the metabolic parameters was obtained.

A series of tests was conducted to check whether the genotypes’ frequencies had a statistically significant effect on different metabolic parameters. Statistically significant differences among groups, determined by genotypes, were estimated using the Kruskal–Wallis test. A post-hoc analysis was performed by applying the Wilcoxon rank-sum test for all pairwise comparisons to discover which genotypes caused the particular test to be significant. Multiple linear regression with adjustment for age and sex was used to test the association between the PRSs and baseline metabolic parameters.

After that, another multiple linear regression, with adjustments for age, sex, and BMI, was used to test the association among the PRSs and the changes in metabolic parameters between baseline and follow-up; a subsequent analysis was carried out but this time with a gender stratification. β coefficients were presented as an incremental increase or decrease in the trait per the SD of the tested PRS. For all the tests described in this section, the *p*-values were adjusted to <0.05 using the false discovery rate correction for multiple comparisons. All calculations were prepared in R (version 4.1.0) [32].

## 4. Discussion

Pathophysiological heterogeneity exists on prediabetes before diagnosis of T2D; therefore, it is essential to develop new methodologies to identify patients with an increased risk of complication without progression to overt T2D. From the PolReD study, 446 subjects who were prediabetic but did not have a diagnosis of T2D at baseline, were selected. The median age of the borderline diabetes cohort is consistent with previous findings showing that prediabetes is more common in middle age (25–44 years), and diabetes is more common in the 45–60 year age group [33], showing that the prevalence of diabetes increases with age. The median values of fasting glucose and HbA1C are 101 mg/dL and 5.76%, respectively. The results are in accordance with the Polish Diabetes Association and American Diabetes Association, that defined prediabetes as the presence of IFG (100–125 mg/dL) and/or HbA1C (5.7–6.4%). IFG identified 274 patients, and HbA1c identified 307, with an overlap of 135 patients (30.3%), consistent with what has been reported in the literature [34].

Following the guidelines published in 2020 [29], the construction of PRSs for T2D and obesity using genome-wide significant variants found in GWAS for T2D and obesity in PolRed’s data was achieved. The methodology followed provided, as a result, weighted PRSs, which have been described as optimal compared with not-weighted PRSs [35]. An ideal PRS should follow a distribution that is symmetrical around its mean, with most values near the central peak. The normal distribution of the two PRSs in the borderline diabetic cohort was confirmed and is shown in two graphics with their mean and standard deviation. We also demonstrated that combining individual variants into a PRS can provide extra information on patterns of T2D susceptibility. Prediabetes and obesity are global pandemics, and their mortality and morbidity are increasing quickly. Obesity is a major contributor to the development of T2D; therefore, there is a close relationship between them. In our study, we established the associations of a T2D PRS and an obesity PRS versus the changes in time in metabolic parameters among a prediabetic Polish-origin Caucasian cohort.

After comparing the parameters at baseline vs. follow-up using the Wilcoxon signed-rank test for paired samples, it was found that 14 of the 18 parameters were statistically significant after adjustment for multiple testing. The increase of the values on the metabolic parameters is expected, as studies have shown that with aging, metabolic disturbances progress, especially when they are not treated, as in the case of our cohort [36]. The difference observed on the IPAQ parameter does not add up to the value increase of the other parameters. The reliability and validity of the IPAQ have been questioned before. The most up-to-day research showed excellent test reliability (reproducible), but low-to-fair concurrent validity for moderate and vigorous physical activity [37]. A better understanding of the associations between exercise and the other metabolic parameters should be performed; moreover, as the questionnaire is a self-reported measure, the results from the IPAQ should be taken with caution, and when possible, compared with results obtained from an accelerometer. The four parameters that are not statistically significant were Chol, Tg, Hdl, and Ldl. When comparing the values of the cohort with the guidelines of lipid profile, we can describe them as optimal for Chol, Tg, and Hdl and above optimal for Ldl [38]. When these parameters’ values are imbalanced with the normal ones, the patient can suffer from dyslipidemia [39]. Lipid profile in prediabetes has been of research relevance recently. In contrast with our data, several studies on the Asian population concluded that prediabetics had a deranged lipid profile compared with normal healthy subjects [40]. This gives an exciting lead to continue studying lipids and their association with prediabetes in European cohorts. Even though the metabolic parameters analyzed in the article are well-known risk factors of T2D, incorporating other parameters that have been associated with the risk of T2D could lead us to discover new associations, adding a great value and possible clinical implications. The risk of developing T2D in a prediabetic cohort has been strongly associated with high blood pressure [41], family history for T2D [42], and history of gestational diabetes [43].

Individuals with high T2D scores exhibited increases in Δ FM. With every SD increase in the PRS, the Δ FM increased by 0.0049 kg. The findings were consistent with previously reported data [44]. In 2021, associations of changes in body weight and FM with incident prediabetes were detected among African Americans and European Americans. Using a linear regression model, they found that a change in FM was a significant predictor of progression in a prediabetes cohort [45]. Furthermore, in 2022, a study on the correlation of prediabetes and T2D with adiposity in adults was reported. On the basis of a multivariable linear regression model, the researchers found an association between adults with prediabetes and increased FM [10]. In contrast with our findings, it has been studied in an animal model that the progression of prediabetes to diabetes is slowed by the increase over time in exercise [46]. Thus, a further research on humans focusing on the Δ IPAQ values will be of great interest. The obesity PRS was associated with the Δ FM and Δ 2 h glucose. For every SD increase in the PRS, the Δ FM and Δ 2 h glucose increased by 0.0056 kg and 0.0013 mg/dL, respectively. Obesity has become a significant problem because of the increase in patients and several metabolic complications. The gene FTO was the first gene identified (through a GWAS) that linked FM and obesity in humans [47]. A few MC4R genetic variants have been associated with FM, weight, and obesity risk [48]. From the 21 SNPs included in our study, seven have a locus on those genes; therefore, the association shown in this study is consistent with previous publications. A change in time of 2 h glucose values has been associated with the development of metabolic diseases such as obesity, T2D, and cardiovascular disease [49] in patients with T2D and prediabetes [50]. Both of our PRSs were associated with changes in FM, which could be related to the overlap of 20 SNPs between the T2D and obesity scores. Studies seeking to associate PRS and other parameters for T2D risk prediction on a prediabetic cohort have been previously conducted. A T2D PRS was found to have an association with age, helping the prediction of early onset T2D [51]. An association of T2D PRS and metabolite data led to enhanced T2D risk prediction by capturing the development of distinct etiologies [52]. Yet another T2D PRS was associated with an oxidative stress score and BMI, with prediction ability for the incidence of prediabetes and T2D [53].

The beta coefficient is the degree of change in the outcome variable for every 1-unit change in the predictor variable (PRS). Statistically, according to the beta coefficient and the *p*-value, the association found is significant and has a positive association. Nevertheless, statistical significance does not always imply practical significance. While a *p*-value can inform whether an effect exists, it will not reveal the size of the effect. Both the adjusted effect size (adjusted r-squared) and statistical significance (*p*-value) are essential results in a multiple linear regression [54]. The association of the T2D PRS and Δ metabolic parameters produced a resulting adjusted r-squared value of 0.10369, meaning that Δ FM explains 10.5% of the variance in PRS; for the association of the obesity PRS and Δ metabolic parameters, the value of the r-squared was 0.21025, meaning that Δ FM and Δ 2 h glucose explains 20% of the variance in PRS. The effect sizes are statistically small; nevertheless, medical research is often associated with small effect sizes in the 0.05 to 0.2 range [54,55]. Despite being small, these effects often represent meaningful effects [55].

We observed significant associations of MTCH2 rs10838738 with the metabolic parameters of the studied group. On the basis of the data, we noted that homozygous AA genotype carriers of rs10838738 presented a significantly greater change in 2 h glucose, while the homozygous GG genotype had a significantly lower change in 2 h insulin. In contrast with our results, previous studies showed that the homozygous genotype of the risk allele G does not have a significant association with 120 min glucose changes, but it does for a high BMI [56]. The gene MTCH2 assists in the recruitment of the BH3 interacting-domain death agonist into the mitochondria during apoptosis [57]. The SNP rs10838738 has been reported to influences insulin sensitivity [58] and impact gene expression in visceral adipose tissue [59]. Nevertheless, as shown in our study, as well as in various previous studies [60], there is little evidence of a significant association of this SNP with diabetes. Extended research must be carried out to discover and demonstrate the impact of this SNP on T2D development. We observed a tendency on PRRC2A rs2260000 according to the metabolic parameters of the studied group. On the basis of our data, we noted that the homozygous of the alternative allele GG genotype carriers presented small changes in FM and VF. The gene PRRC2A plays a role in the regulation of pre-mRNA splicing [61]. No data have been published regarding an association between PRRC2A rs2260000 and T2D. Nonetheless, recent studies have associated variants of the gene PRRC2A with obesity [62] and type 1 diabetes risk [63]. Furthermore, in accordance with our results, recent data demonstrate a relation between this gene and human adipocytes isolated from VF [64]. An increase in publications citing the function of PRRC2A has been noted since 2020; therefore, a tendency for its research is clearly observed. We observed a tendency on ETV5 rs7647305, according to the metabolic parameters of the studied group. On the basis of the data, we noted that the homozygous of the alternative allele CC genotype carriers presented significantly great changes in 2 h glucose and significantly small changes on IPAQ. Associations between this gene and T2D have been described before; the ETV5 influences b-cell dysfunction and the pathophysiology of T2D [65]. As well, the critical role of ETV5 in regulating insulin secretion has been demonstrated [66], as has the importance of studying this gene to compare sedentary behavior versus physical activity [67]. A tendency on KCTD15 rs29941 was observed, according to the metabolic parameters of the studied group. On the basis of the data, we noted that AG genotype carriers presented significantly great changes in the 2 h glucose. KCTD15 enables identical protein-binding activity [68]. The gene KCTD15 rs29941 has been previously associated with the risk of obesity, fasting glucose, insulin resistance, and T2D [69].

The present study has several strengths. As far as we know, this is one of the first studies to research the associations of the changes over time in several metabolic parameters with T2D and obesity PRSs in a prediabetic Polish population. Another strength of our study is that it is based on a relatively large population. The established associations between PRSs and changes in metabolic traits related to T2D and obesity show how the genetic information of patients with prediabetes could help prediction. Knowing the status of risk within the prediabetic cohort will also help prevent adjacent complications included in the metabolic syndrome, such as hypertension, obesity, and heart disease. Finally, while the risk factors for T2D are known, predictive markers that have the power of classifying a prediabetic population into subgroups according to their level of risk are still not developed. Combining the associations found into a clustering analysis with other parameters previously described as highly relevant for T2D risk prediction in a prediabetic cohort [24] would translate our results into clinical evidence.

Nevertheless, several limitations of our study also need to be addressed. For one, there is a clear need for more long-term human studies to evaluate how Δ 2 h glucose is associated with the different genotypes described in our study. A better understanding is also needed of the lack of association between the changes over time in the other metabolic parameters and our PRSs. Moreover, only Caucasian individuals were recruited for our study, and the effect size estimates used to create the PRSs were based on European ancestry. As such, this study did not consider the possibility of different effect sizes for different populations. Therefore, the data should be replicated in other cohorts to verify and validate our findings.

## 5. Conclusions

Our findings suggest that there is some overlap between genes implicated in the risk of developing T2D and those associated with the risk of obesity. It was found that the Δ FM is associated with T2D and obesity PRSs in a prediabetic cohort and that the Δ glucose at 120 min is associated with obesity’s PRS. These findings are consistent with recent results demonstrating that an increase in the change of FM and obesity are closely related to insulin resistance and abnormalities in glucose metabolism and, therefore, T2D risk.

Although metabolic traits and genetic variants have been identified and associated, their interpretations and translations into clinical practice need to be further investigated by adding more data and clinical trials. The results should be taken with caution, as more associations need to be found, and validation in larger populations and among different ethnic groups needs to be conducted before any clinical implications. The associations found in this research could be considered a pilot study for producing a powerful tool. The strategy will stratify prediabetic individuals according to their risk of developing T2D and obesity and help to guide the early treatment and prevention of the diseases, thereby defining the first step towards precision medicine for prediabetes and T2D.

## Figures and Tables

**Table 1:** Description of cohort parameters and comparison in the prediabetes (n = 446) cohort between baseline and the follow-up.

Characteristics and Parameters	Baseline	Follow-Up	*p*-Value
	Median (IR)	Median (IR)	
Female (n%)	245 (54.9%)		-
Age (years)	42.54 (30.33, 55.73)	47.43 (35.63, 60.67)	3.16 × 10^−24^
BMI (kg/m^2^)	26.87 (24.04, 30.85)	27.51 (24.36, 31.780)	7.64 × 10^−13^
FFM (kg)	53.92 (48.74, 61.52)	50.74 (44.55, 62.16)	1.13 × 10^−21^
FM (kg)	23.25 (20.06, 28.23)	26.82 (21.22, 36.14)	1.73 × 10^−24^
MM (kg)	24.98 (21.17, 30.3)	24.85 (21.15, 32.58)	0.0010
VF (cm^3^)	82.50 (65, 101)	112 (72.25, 152)	3.94 × 10^−15^
SF (cm^3^)	145.50 (117, 184)	249 (180.25, 318.75)	1.32 × 10^−45^
VAT/SAT ratio	0.54 (0.44, 0.64)	0.42 (0.33, 0.56)	3.82 × 10^−13^
IPAQ (min/week)	1344 (240, 4306)	5368 (2530, 11546)	3.90 × 10^−24^
Fasting glucose (mg/dL)	101 (95, 110)	109 (98, 121)	8.10 × 10^−46^
2 h glucose (mg/dL)	120 (103, 131)	129 (111.25, 139.75)	1.50 × 10^−14^
HbA1c (%)	5.76 (5.51, 6.00)	5.82 (5.59, 6.10)	0.0002
Fasting insulin (uU/mL)	10.78 (8.50, 14.75)	11.87 (9.40, 15.67)	0.0006
2 h insulin (uU/mL)	29.98 (17.17, 53.84)	34.11 (22.23, 49.89)	0.0154
Chol (mg/dL)	188 (165, 221)	194 (169, 220)	0.0726
Tg (mg/dL)	91 (67.25, 133.00)	96.90 (71.25, 143.00)	0.0918
Hdl (mg/dL)	59.70 (50.42, 68.00)	57.5 (47, 70)	0.2003
Ldl (mg/dL)	109.60 (83.25, 137.40)	108.80 (87.40, 138.30)	0.0760

IR: interquartile range; BMI: body mass index; FFM: fat-free mass; FM: fat mass; MM: muscle mass; VF: visceral fat; SF: subcutaneous fat; VAT: visceral adipose tissue; SAT: subcutaneous adipose tissue; IPAQ: International Physical Activity Questionnaire; Hba1c: glycated hemoglobin; Chol: total cholesterol; Tg: triglycerides; Hdl: high-density lipoprotein; Ldl: low-density lipoproteins. *p*-values of 0.05 are in bold and reflect significance after adjustment for multiple testing.

**Table 2:** Association of T2D PRS with changes in metabolic parameters after follow-ups with the prediabetic cohort in PolRed.

Metabolic Parameter	β (95% CI)	*p*-Value
Δ FFM (kg)	0.0017 (−0.0029, 0.0063)	0.462
Δ FM (kg)	0.0049 (−0.0006, 0.0091)	**0.025**
Δ MM (kg)	0.0001 (−0.0004, 0.0002)	0.548
Δ VF (cm^3^)	0.0001 (−0.0009, 0.0012)	0.802
Δ SF (cm^3^)	0.0001 (−0.0004, 0.0006)	0.738
Δ VAT/SAT ratio	−0.0369 (−0.1955, 0.1216)	0.647
Δ IPAQ (min/week)	0.0001 (−0.0004, 0.0002)	0.269
Δ Fasting glucose (mg/dL)	−0.0010 (−0.0034, 0.0013)	0.394
Δ 2 h glucose (mg/dL)	−0.0004 (−0.0015, 0.0007)	0.467
Δ HbA1c (%)	0.0492 (−0.0242, 0.1226)	0.188
Δ Fasting insulin (uU/mL)	0.0011 (−0.0024, 0.0045)	0.548
Δ 2 h insulin (uU/mL)	0.0002 (−0.008, 0.0012)	0.650
Δ Chol (mg/dL)	0.0063 (−0.0133, 0.0259)	0.531
Δ Tg (mg/dL)	−0.0013 (−0.0053, 0.0026)	0.507
Δ Hdl (mg/dL)	−0.0066 (−0.0261, 0.0130)	0.511
Δ Ldl (mg/dL)	−0.0065 (−0.0261, 0.0132)	0.518

Δ: Difference of the metabolic parameter at their T2 vs. T1; FFM: fat-free mass; FM: fat mass; MM: muscle mass; VF: visceral fat; SF: subcutaneous fat; VAT: visceral adipose tissue; SAT: subcutaneous adipose tissue; IPAQ: International Physical Activity Questionnaire; Hba1c: glycated hemoglobin; Chol: total cholesterol; Tg: triglycerides; Hdl: high-density lipoprotein; Ldl: low-density lipoproteins. β values are reported per SD of polygenic score. *p*-values of 0.05 are in bold and reflect significance after adjustment for multiple testing.

**Table 3:** Association of obesity PRS with changes in metabolic parameters after follow-up in PolRed.

Metabolic Parameter	β (95% CI)	*p*-Value
Δ FFM (kg)	0.0021 (−0.0032, 0.0074)	0.4383
Δ FM (kg)	0.0056 (−0.0008, 0.0105)	**0.0231**
Δ MM (kg)	0.0002 (−0.0002, 0.0005)	0.3184
Δ VF (cm^3^)	0.0002 (−0.0002, 0.0005)	0.7600
Δ SF (cm^3^)	0.0001 (−0.006, 0.0006)	0.8850
Δ VAT/SAT ratio	0.0205 (−0.1619, 0.2029)	0.8252
Δ IPAQ (min/week)	0.0001 (−0.0004, 0.0002)	0.4108
Δ Glucose time 0 (mg/dl)	0.0020 (−0.0007, 0.0047)	0.1446
Δ 2 h glucose (mg/dl)	0.0013 (−0.0001, 0.0026)	**0.0341**
Δ HbA1c (%)	0.0014 (−0.0830, 0.0859)	0.9732
Δ Fasting insulin (uU/mL)	−0.0024 (−0.0064, 0.0016)	0.2316
Δ 2 h insulin (uU/mL)	0.0007 (−0.0004, 0.0018)	0.2273
Δ Chol (mg/dL)	0.0168 (−0.0057, 0.0394)	0.1434
Δ Tg (mg/dL)	−0.0036 (−0.0081, 0.0009)	0.1167
Δ Hdl (mg/dL)	−0.0158 (−0.0383, 0.0067)	0.1685
Δ Ldl (mg/dL)	−0.0164 (−0.0390, 0.0061)	0.1533

Δ: Difference of the metabolic parameter at their T2 vs. T1. FFM: fat-free mass; FM: fat mass; MM: muscle mass; VF: visceral fat; SF: subcutaneous fat; VAT: visceral adipose tissue; SAT: subcutaneous adipose tissue; IPAQ: International Physical Activity Questionnaire; Hba1c: glycated hemoglobin; Chol: total cholesterol; Tg: triglycerides; Hdl: high-density lipoprotein; Ldl: low-density lipoproteins. β values are reported per SD of polygenic score. *p*-values of 0.05 are in bold and reflect significance after adjustment for multiple testing.

**Table 4:** Description and comparison of the prediabetic cohort participants stratified by rs10838738, rs2260000, rs7647305, and rs29941 genotypes.

rs10838738 (*MTCH2)*	A/A	A/G	G/G	*p*-value
N	143	229	83	
Δ 2 h glucose (mg/dL)	19 (3, 35)	13 (−6.75, 33)	8 (−8, 23.5)	0.017
Δ 2 h insulin (uU/mL)	5.88 (−5.85, 21.34)	3.74 (−10.25, 22.49)	−1.68 (−26.02, 10.02)	0.001
rs2260000 (*PRRC2A)*	A/A	A/G	G/G	*P§*
N	151	223	81	
Δ FM (kg)	5.27 (0.50, 10.17)	4.87 (1.32, 8.96)	2.40 (−1.92, 6.45)	0.051
Δ VF (cm3)	35.75 (−8.25, 73)	29 (−6, 67)	8 (−29.50, 53.50)	0.068
rs7647305 (*ETV5)*	C/C	C/T	T/T	*P§*
N	309	126	20	
Δ 2 h glucose (mg/dl)	13.3 (−1, 35)	10.5 (−11.8, 25)	9 (−30, 27.5)	0.077
Δ IPAQ (min/week)	2712.5 (−604.9, 6962.2)	2601 (−23.3, 9537.3)	6463 (2470, 12,901)	0.077
rs29941 (*KCTD15)*	A/A	A/G	G/G	*P§*
N	38	204	213	
Δ 2 h glucose (mg/dL)	20.5 (12, 33)	15 (0.75, 36.25)	9.50 (−9.50, 26.25)	0.068

Data presented as median and interquartile range, number of observations, and frequency. Δ: Difference of the metabolic parameter at their T2 vs. T1; FM: fat mass; VF: visceral fat; IPAQ: International Physical Activity Questionnaire. *p*-values after adjustment for multiple testing.

## Data Availability

The data that support the findings of this study are available on request from the corresponding author, F.P.-M. The data are not publicly available due to containing information that could compromise the privacy of research participants.

## Supplementary Materials

The following supporting information can be downloaded at: https://www.mdpi.com/article/10.3390/ijms232416081/s1, Supplementary Table S1. Genetic variants included in the T2D PRS; Supplementary Table S2. Genetic variants included in the Obesity PRS; Supplementary Table S3. Association of T2D PRS with the baseline metabolic parameters in PolRed; Supplementary Table S4. Association of Obesity PRS with the baseline metabolic parameters in PolRed; Supplementary Table S5. Association of T2D PRS with changes in metabolic parameters with gender stratification; Supplementary Table S6. Association of Obesity PRS with changes in metabolic parameters with gender stratification.
