# Supplementary material for: Testing the Utility of Polygenic Risk Scores for Type 2 Diabetes and Obesity in Predicting Metabolic Changes in a Prediabetic Population: An Observational Study
Source: Int J Mol Sci. 2022 Dec 16;23(24):16081. doi: 10.3390/ijms232416081 (PMC9787993; doi:10.3390/ijms232416081)
Supplement: Supplementary file 1 [file ijms-23-16081-s001.zip › ijms-2043324-supplementary.pdf]

SUPPLEMENTARY MATERIAL

Supplementary Table S1. Genetic variants included in the T2D PRS.

SNP	Locus	CHR	Pos	Alt Allele	β	Odd Ratio	MAF	P-value	N
rs3101336	*NEGR1*	1	72751185	C	0.018	1.018	0.450	3.07E-02	939912
rs2568958	*NEGR1*	1	72765116	A	0.022	1.022	0.438	1.64E-04	1082380
rs2815752	*NEGR1*	1	72812440	A	0.022	1.022	0.388	4.05E-04	1084120
rs10913469	*SEC16B*	1	177913519	C	0.032	1.033	0.163	1.74E-12	1073630
rs340874	*PROX1*	1	214159256	C	0.047	1.048	0.475	6.11E-32	1081640
rs2605100	*LYPLAL1*	1	219644224	G	0.030	1.031	0.213	3.75E-07	1090350
rs12022722	*LYPLAL1*	1	219651133	T	0.020	1.020	0.413	7.57E-06	917427
rs2820464	*LYPLAL1*	1	219693220	A	-0.029	0.972	0.175	4.82E-10	927501
rs2785980	*LYPLAL1*	1	219700519	C	-0.031	0.969	0.175	1.30E-13	1039880
rs4846567	*SLC30A10*	1	219750717	T	-0.033	0.967	0.125	1.17E-15	1078840
rs6548238	*TMEM18*	2	634905	C	0.054	1.055	0.113	1.66E-21	1083220
rs7561317	*TMEM18*	2	644953	G	0.056	1.058	0.238	2.67E-22	1023140
rs780094	*GCKR*	2	27741237	C	0.053	1.055	0.488	2.05E-34	1082280
rs13389219	*GRB14*	2	165528876	T	-0.054	0.947	0.400	5.98E-35	1080230
rs7607980	*GRB14*	2	165551201	C	-0.068	0.935	0.138	1.75E-19	851502
rs1801282	*PPARG*	3	12393125	G	-0.022	0.978	0.263	4.85E-02	18252
rs11708067	*ADCY5*	3	123065778	G	-0.072	0.930	0.100	1.06E-44	756464
rs11920090	*SLC2A2*	3	170717521	A	-0.027	0.974	0.188	1.47E-03	952836
rs4402960	*IGF2BP2*	3	185511687	T	0.096	1.101	0.425	4.15E-122	1088690
rs7647305	*ETV5*	3	185834290	C	0.026	1.027	0.213	7.70E-07	965163
rs10938397	*GNPDA2*	4	45182527	G	0.038	1.039	0.388	5.11E-17	1048050
rs10946398	*CDKAL1*	6	20661034	C	0.113	1.120	0.375	5.97E-144	1085220
rs2844479	*AIF1*	6	31572956	C	0.026	1.027	0.500	4.49E-09	1078460
rs2260000	*PRRC2A*	6	31593476	G	0.020	1.020	0.375	2.71E-05	930840
rs1077393	*BAG6*	6	31610529	G	0.019	1.019	0.500	1.41E-04	1081460
rs2191349	*DGKB*	7	15064309	T	0.051	1.053	0.488	1.23E-41	1081160
rs4607517	*YKT6*	7	44235668	A	0.032	1.032	0.088	2.65E-10	1082060
rs4731702	*KLF14*	7	130433384	T	-0.037	0.964	0.475	1.09E-20	1075480
rs972283	*KLF14*	7	130466854	G	0.035	1.036	0.463	5.05E-20	1067780
rs13266634	*SLC30A8*	8	118184783	T	-0.091	0.913	0.388	2.45E-124	1181190
rs11558471	*SLC30A8*	8	118185733	G	-0.088	0.916	0.313	8.78E-106	1082770
rs7034200	*GLIS3*	9	4289050	A	0.040	1.041	0.488	1.20E-23	1070410
rs10811661	*GLIS3*	9	22134094	C	-0.140	0.869	0.113	2.88E-146	930908
rs1111875	*HHEX*	10	94462882	T	-0.093	0.912	0.388	7.30E-95	938378
rs5015480	*HHEX*	10	94465559	T	-0.089	0.915	0.388	1.64E-128	1069430
rs7923837	*HHEX*	10	94481917	A	-0.094	0.911	0.350	1.62E-75	888616
rs10885122	*ADRA2A*	10	113042093	G	0.024	1.024	0.125	1.03E-05	1082070
rs7901695	*TCF7L2*	10	114754088	C	0.207	1.230	0.313	5.82E-226	209644

rs4506565	*TCF7L2*	10	114756041	T	0.216	1.241	0.313	6.14E-229	208956
rs7903146	*TCF7L2*	10	114758349	T	0.235	1.265	0.275	2.64E-97	249463
rs5215	*KCNJ11*	11	17408630	T	-0.056	0.945	0.363	5.74E-52	1192080
rs4074134	*BDNF*	11	27647285	T	-0.023	0.978	0.150	1.59E-05	937741
rs4923461	*BDNF*	11	27656910	G	-0.019	0.981	0.150	3.05E-05	931716
rs925946	*BDNF*	11	27667202	G	0.000	1.000	0.363	2.44E-05	951966
rs10501087	*BDNF*	11	27670108	C	-0.023	0.977	0.150	2.81E-06	934,105
rs6265	*BDNF*	11	27679916	T	-0.022	0.978	0.138	9.00E-07	1169040
rs10835211	*BDNF*	11	27701365	A	0.022	1.103	0.213	1.83E-02	818173
rs11605924	*CRY2*	11	45873091	C	-0.021	0.979	0.450	4.96E-08	964253
rs10838738	*MTCH2*	11	47663049	G	0.013	1.013	0.363	5.75E-03	1085580
rs174550	*FADS1*	11	61571478	C	-0.027	0.973	0.350	1.20E-10	1019910
rs10830963	*MTNR1B*	11	92708710	G	0.061	1.063	0.225	7.36E-43	1059540
rs7138803	*BCDIN3D*	12	50247468	A	0.023	1.023	0.425	1.35E-09	1089630
rs8049439	*ATXN2L*	16	28837515	C	0.019	1.019	0.413	2.76E-05	1191610
rs4788102	*SH2B1*	16	28873398	A	0.022	1.022	0.413	8.70E-06	933067
rs7498665	*SH2B1*	16	28883241	G	0.022	1.023	0.400	1.86E-06	1024050
rs6499640	*FTO*	16	53769677	A	0.027	1.027	0.425	5.97E-08	1080780
rs8050136	*FTO*	16	53816275	A	0.094	1.098	0.488	8.61E-102	1090360
rs3751812	*FTO*	16	53818460	T	0.096	1.100	0.488	2.47E-76	935802
rs9939609	*FTO*	16	53820527	A	0.092	1.096	0.488	5.44E-131	1063990
rs7190492	*FTO*	16	53828752	G	0.048	1.049	0.325	8.44E-18	937716
rs8044769	*FTO*	16	53839135	C	0.061	1.062	0.438	2.73E-31	937714
rs633265	*MC4R*	18	57831468	T	0.039	1.040	0.413	1.05E-18	931300
rs1350341	*MC4R*	18	57842533	A	0.038	1.039	0.400	5.37E-18	926826
rs17782313	*MC4R*	18	57851097	C	0.055	1.057	0.238	1.46E-35	1088670
rs12970134	*MC4R*	18	57884750	A	0.050	1.051	0.300	2.83E-28	1089600
rs29941	*KCTD15*	19	34309532	G	0.001	1.001	0.338	1.36E-04	1082030
rs11084753	*KCTD16*	19	34322137	G	0.011	1.011	0.363	2.61E-03	1047670
rs2287019	*QPCTL*	19	46202172	T	0.021	1.021	0.213	1.00E-04	1149250

T2D = type 2 diabetes, PRS = polygenic risk-score SNP = single nucleotide polymorphism, CHR = , Pos = position based on human genome 19, MAF = minor allele frequency, P value = statistical significant associations of SNPS in GWAS for type 2 diabetes (https://t2d.hugeamp.org/), N = Effective sample size from all the dataset where the P-value was significant**.**

Supplementary Table S2. Genetic variants included in the Obesity PRS

SNP	Locus	CHR	Pos	Alt Allele	β	Odd Ratio	MAF	P-value	N
rs7561317	*TMEM18*	2	644953	G	0.141	1.151	0.2451	1.38E-04	11743
rs10938397	*GNPDA2*	4	45182527	G	0.099	1.104	0.4549	2.60E-04	11743
rs7903146	*TCF7L2*	10	114758349	T	-0.101	0.904	0.2280	3.16E-03	11743
rs4074134	*BDNF*	11	27647285	T	-0.163	0.850	0.2280	7.37E-06	11743
rs4923461	*BDNF*	11	27656910	G	-0.163	0.850	0.2232	6.98E-06	11743
rs925946	*BDNF*	11	27667202	G	-0.088	0.915	0.2610	1.88E-03	11743
rs10501087	*BDNF*	11	27670108	C	-0.162	0.850	0.2220	8.07E-06	11743
rs6265	*BDNF*	11	27679916	T	-0.164	0.849	0.2061	1.17E-05	11743
rs10835211	*BDNF*	11	27701365	A	0.098	1.103	0.1915	2.04E-03	11743
rs8049439	*ATXN2L*	16	28837515	C	0.087	1.091	0.4305	1.39E-03	11743
rs4788102	*SH2B1*	16	28873398	A	0.094	1.099	0.4256	5.71E-04	11743
rs7498665	*SH2B1*	16	28883241	G	0.094	1.099	0.4207	5.65E-04	11743
rs8050136	*FTO*	16	53816275	A	0.205	1.228	0.4915	9.23E-14	11743
rs3751812	*FTO*	16	53818460	T	0.206	1.229	0.4902	6.42E-14	11743
rs9939609	*FTO*	16	53820527	A	0.207	1.231	0.4890	5.07E-14	11743
rs7190492	*FTO*	16	53828752	G	0.126	1.134	0.3232	5.28E-06	11743
rs8044769	*FTO*	16	53839135	C	0.162	1.176	0.4195	1.76E-09	11743
rs9921518	*IRX3*	16	54494424	G	-0.105	0.900	0.2488	6.11E-03	11743
rs17782313	*MC4R*	18	57851097	C	0.102	1.107	0.1768	3.42E-03	11743
rs12970134	*MC4R*	18	57884750	A	0.099	1.104	0.2134	3.66E-03	11743
rs29941	*KCTD15*	19	34309532	G	0.061	1.063	0.3073	2.77E-02	11743

PRS = polygenic risk-score SNP = single nucleotide polymorphism, CHR = , Pos = position based on human genome 19, MAF = minor allele frequency, P value = statistical significant associations of SNPS in GWAS for Obesity (https://t2d.hugeamp.org/), N = Effective sample size, DataSet: FinnGen 2018 GWAS: European ancestry (https://t2d.hugeamp.org/dinspector.html?dataset=GWAS_FinnGen_eu&phenotype=Obesity).

Supplementary Table S3. Association of T2D PRS with the baseline metabolic parameters in PolRed.

**Metabolic Parameter**	**β (95% CI)**	***P*§**
BMI (kg/m^2)	0.0066 (-0.0020, 0.0112)	**0.0053**
FFM (kg)	0.0029 (-0.0001, 0.0056)	**0.0394**
FM (kg)	0.0028 (-0.0003, 0.0053)	**0.0293**
MM (kg)	-0.0045 (-0.0091, 0.0001)	0.0535
VF (cm^3)	-0.0004 (-0.0009, 0.0001)	0.0662
SF (cm^3)	-0.0003 (-0.0007, 0.0001)	0.1360
VAT/SAT ratio	-0.0544 (-0.1436, 0.0348)	0.2310
IPAQ (min/week)	0.0002 (-0.0002, 0.0005)	0.4450
Fasting glucose (mg/dl)	0.0024 (-0.0001, 0.0047)	**0.0451**
2-hour glucose (mg/dl)	0.0001 (-0.0008, 0.0009)	0.8990
HbA1c (%)	-0.0171 (-0.0629, 0.0288)	0.4650
Fasting insulin (uU/ml)	0.0033 (-0.0003, 0.0063)	**0.0320**
2-hour insulin (uU/ml)	-0.0001 (-0.0008, 0.0006)	0.7740
Chol (mg/dL)	0.3426 (-0.8705, 1.5558)	0.5790
Tg (mg/dL)	-0.0683 (-0.3109, 0.1744)	0.5800
Hdl (mg/dL)	-0.3415 (-1.5546, 0.8716)	0.5800
Ldl (mg/dL)	-0.3428 (-1.556, 0.8704)	0.5790

BMI: Body mass index; FFM: fat free mass; FM: fat mass; MM: muscle mass; VF: visceral fat; SF: subcutaneous fat; VAT: visceral adipose tissue; SAT: subcutaneous adipose tissue; IPAQ: International Physical Activity Questionnaire; Hba1c: glycated hemoglobin; Chol: total cholesterol; Tg: triglycerides; Hdl: high-density lipoprotein; Ldl: low-density lipoproteins. β values are reported per SD of polygenic score. *P*§ values of 0.05 are in bold and reflect significance after adjustment for multiple testing.

Supplementary Table S4. Association of Obesity PRS with the baseline metabolic parameters in PolRed.

Metabolic Parameter	β (95% CI)	*P*§
BMI (kg/m^2)	0.0141 (-0.0028, 0.0255)	**0.0145**
FFM (kg)	0.0027 (-0.0259 , 0.0312)	0.8539
FM (kg)	0.0043 (-0.0039 , 0.0126)	0.3016
MM (kg)	-0.0057 (-0.0522 , 0.0409)	0.8111
VF (cm^3)	0.0013 (-0.0006 , 0.0032)	0.1773
SF (cm^3)	-0.0008 (-0.0020 , 0.0005)	0.2489
VAT/SAT ratio	-0.2228 (-0.4980 , 0.0523)	0.1121
IPAQ (min/week)	-0.0001 (-0.0006 , 0.0003)	0.5656
Fasting glucose (mg/dl)	-0.0033 (-0.0073 , 0.0006)	0.0978
2-hour glucose (mg/dl)	0.0003 (-0.0012 , 0.0019)	0.6857
HbA1c (%)	0.0371 (-0.0256 , 0.0998)	0.2449
Fasting insulin (uU/ml)	0.0017 (-0.0029 , 0.0063)	0.4560
2-hour insulin (uU/ml)	0.0004 (-0.0009 , 0.0017)	0.5303
Chol (mg/dL)	0.8328 (-0.5718 , 2.2374)	0.2445
Tg (mg/dL)	-0.1660 (-0.4469 , 0.1149)	0.2460
Hdl (mg/dL)	-0.8329 (-2.2374 , 0.5716)	0.2444
Ldl (mg/dL)	-0.8327 (-2.2373 ,0.5720)	0.2445

BMI: Body mass index; FFM: fat free mass; FM: fat mass; MM: muscle mass; VF: visceral fat; SF: subcutaneous fat; VAT: visceral adipose tissue; SAT: subcutaneous adipose tissue; IPAQ: International Physical Activity Questionnaire; Hba1c: glycated hemoglobin; Chol: total cholesterol; Tg: triglycerides; Hdl: high-density lipoprotein; Ldl: low-density lipoproteins. β values are reported per SD of polygenic score. *P*§ values of 0.05 are in bold and reflect significance after adjustment for multiple testing.

Supplementary Table S5. Association of T2D PRS with changes in metabolic parameters with gender stratification.

Metabolic Parameter	*Female (n = 245)*		*Male (n = 201)*	
	**β (95% CI)**	***P§***	**β (95% CI)**	***P§***
FFM (kg)	0.0047 (-0.0032, 0.0126)	0.2453	-0.0016(-0.0121, 0.0087)	0.750
FM (kg)	0.0068 (-0.0013, 0.0123)	**0.0158**	0.0024 (0-.0040, 0.0089,)	**0.045**
MM (kg)	-0.0001 (-0.0004, 0.0002)	0.5088	0.0069 (-0.0079, 0.02180)	0.361
VF (cm^3)	-0.0006 (-0.0020, 0.0008)	0.4129	0.0009 (-0.0005, 0.0023)	0.214
SF (cm^3)	0.0004 (-0.0002, 0.0012)	0.1966	-0.0003 (-0.0010, 0.0004)	0.397
VAT/SAT ratio	0.0720 (-0.1583, 0.3023)	0.5384	-0.1753 (-0.4043, 0.05384)	0.133
IPAQ (min/week)	0.0001 (-0.0002, 0.0002)	0.6696	0.0001 (-0.0002, 0.0002)	0.518
Fasting glucose (mg/dl)	-0.0007 (-0.0044, 0.0029)	0.6866	-0.0005 (-0.0036, 0.0025)	0.706
2-hour glucose (mg/dl)	0.0005 (-0.0013, 0.0014)	0.3016	-0.0012 (-0.0030, 0.0005)	0.164
HbA1c (%)	0.105 (0.0081, 0.2018)	0.0937	-0.0011 (-0.1198, 0.1176)	0.985
Fasting insulin (uU/ml)	0.0002 (-0.0041, 0.0045)	0.9248	0.0046 (-0.0004, 0.0097)	0.076
2-hour insulin (uU/ml)	0.0008 (-0.0004, 0.002)	0.202	-0.0008 (-0.0024, 0.0008)	0.328
Chol (mg/dL)	-0.0301 (-0.0798, 0.0195)	0.2337	0.01634 (-0.0055, 0.0382)	0.142
Tg (mg/dL)	0.0056 (-0.0043, 0.01567)	0.2632	-0.0032 (-0.0076, 0.0011)	0.147
Hdl (mg/dL)	0.0278 (-0.0218, 0.0775)	0.2706	-0.0146 (-0.0364, 0.0071)	0.187
Ldl (mg/dL)	0.0298 (-0.0199, 0.0795)	0.2391	-0.016 (-0.0378, 0.0059)	0.151

FFM: fat free mass; FM: fat mass; MM: muscle mass; VF: visceral fat; SF: subcutaneous fat; VAT: visceral adipose tissue; SAT: subcutaneous adipose tissue; IPAQ: International Physical Activity Questionnaire; Hba1c: glycated hemoglobin; Chol: total cholesterol; Tg: triglycerides; Hdl: high-density lipoprotein; Ldl: low-density lipoproteins. β values are reported per SD of polygenic score. *P§* values of 0.05 are in bold and reflect significance after adjustment for multiple testing.

Supplementary Table S6. Association of Obesity PRS with changes in metabolic parameters with gender stratification.

Metabolic Parameter	*Female (n = 245)*		*Male (n = 201)*	
	**β (95% CI)**	***P*§**	**β (95% CI)**	***P*§**
FFM (kg)	0.0020 (-0.0070, 0.0111)	0.657	0.0057 (-0.0064, 0.0180)	0.352
FM (kg)	0.0064 (-0.0001, 0.0128)	**0.045**	0.0049 (-0.0026, 0.0125,)	**0.020**
MM (kg)	0.0001 (-0.0001, 0.00052)	0.293	-0.0035 (-0.0211, 0.0140)	0.690
VF (cm^3)	-0.0005 (-0.0022, 0.00115)	0.536	0.0006 (-0.0010, 0.0023)	0.432
SF (cm^3)	0.0003 (-0.0004, 0.0012)	0.396	-0.0003 (-0.0012, 0.0005)	0.461
VAT/SAT ratio	0.1525 (-0.1114, 0.4163)	0.256	-0.0907 (-0.3592, 0.1778)	0.506
IPAQ (min/week)	-0.0001 (-0.0002, 0.0002)	0.154	0.0001 (-0.0002, 0.0002)	0.787
Fasting glucose (mg/dl)	0.0029 (-0.0013, 0.0071)	0.181	0.0020 (-0.0015, 0.0057)	0.260
2-hour glucose (mg/dl)	0.0004 (-0.0011, 0.0019)	**0.049**	0.0021 (-0.0001, 0.0042,)	**0.044**
HbA1c (%)	0.0851 (-0.0247, 0.1951)	0.128	-0.0939 (-0.2340, 0.0461)	0.187
Fasting insulin (uU/ml)	-0.0039 (-0.0094, 0.0014)	0.154	0.0003 (-0.0056, 0.0063)	0.910
2-hour insulin (uU/ml)	0.0006 (-0.0007, 0.0020)	0.347	0.0001 (-0.0018, 0.0020)	0.908
Chol (mg/dL)	-0.0377 (-0.0942, 0.0187)	0.189	0.0264 (0.0007, 0.0522)	0.044
Tg (mg/dL)	0.0074 (-0.0038, 0.0188)	0.194	-0.0055 (-0.0107, -0.0004)	0.035
Hdl (mg/dL)	0.0391 (-0.01732, 0.0956)	0.173	-0.0260 (-0.0516, -0.0003)	0.047
Ldl (mg/dL)	0.0381 (-0.0183, 0.0947)	0.185	-0.0257 (-0.0515, -0.0001)	0.050

FFM: fat free mass; FM: fat mass; MM: muscle mass; VF: visceral fat; SF: subcutaneous fat; VAT: visceral adipose tissue; SAT: subcutaneous adipose tissue; IPAQ: International Physical Activity Questionnaire; Hba1c: glycated hemoglobin; Chol: total cholesterol; Tg: triglycerides; Hdl: high-density lipoprotein; Ldl: low-density lipoproteins. β values are reported per SD of polygenic score. *P*§ values of 0.05 are in bold and reflect significance after adjustment for multiple testing.
